# Engineering of cyclodextrin glycosyltransferase improves the conversion efficiency of rebaudioside A to glucosylated steviol glycosides and increases the content of short-chain glycosylated steviol glycoside

**DOI:** 10.1186/s12934-023-02121-2

**Published:** 2023-06-14

**Authors:** Ruiqin Zhang, Ruiqi Tang, Wei Wang, Jiahua Bi, Xianrui Xu, Qiuling Fan, Yanjun Li, Qihe Chen

**Affiliations:** 1 Department of Food Science and Nutrition, Zhejiang University, Hangzhou, 310058 China; 2 Key Laboratory of Food and Biological Engineering of Zhejiang Province, Research and Development Department, Hangzhou Wahaha Technology Co. Ltd, Hangzhou Wahaha Group Co. Ltd, Hangzhou, 310018 China; 3 Key Laboratory of Bioprocess Engineering of Jiangxi Province, College of Life Sciences, Jiangxi Science and Technology Normal University, Nanchang, 330013 China; 4 School of Biological and Chemical Engineering, Zhejiang University of Science and Technology, Hangzhou, 310000 China

**Keywords:** Cyclodextrin glucanotransferase, Steviol glycoside glycosylation, Site-directed mutagenesis, Short-chain glycosylated product

## Abstract

**Background:**

Compared with steviol glycosides, the taste of glucosylated steviol glycosides is better and more similar to that of sucrose. At present, cyclodextrin glucanotransferase (CGTase) is primarily used to catalyze the conversion of steviol glycosides to glucosylated steviol glycosides, with soluble starch serving as a glycosyl donor. The main disadvantages of enzymatic transglycosylation are the limited number of enzymes available, the low conversion rates that result in low yields, and the lack of selectivity in the degree of glycosylation of the products. In order to fill these gaps, the proteome of *Alkalihalobacillus oshimensis* (also named *Bacillus oshimensis*) was used for mining novel CGTases.

**Results:**

Here, CGTase-15, a novel β-CGTase with a wide pH adaptation range, was identified and characterized. The catalyzed product of CGTase-15 tasted better than that of the commercial enzyme (Toruzyme® 3.0 L). In addition, two amino acid sites, Y199 and G265, which play important roles in the conversion of steviol glycosides to glucosylated steviol glycosides were identified by site-directed mutagenesis. Compared with CGTase-15, CGTase-15-Y199F mutant significantly increased the conversion rate of rebaudioside A (RA) to glucosylated steviol glycosides. Compared with CGTase-15, the content of short-chain glycosylated steviol glycosides catalyzed by CGTase-15-G265A mutant was significantly increased. Moreover, the function of Y199 and G265 was verified in other CGTases. The above mutation pattern has also been applied to CGTase-13 (a CGTase discovered by our laboratory with great potential in the production of glycosylated steviol glycosides), confirming that the catalytic product of CGTase-13-Y189F/G255A mutant has a better taste than that of CGTase-13.

**Conclusions:**

This is the first report on the improvement of the sensory profiles of glycosylated steviol glycosides through site-directed mutagenesis of CGTase, which is significant for the production of glycosylated steviol glycosides.

**Supplementary Information:**

The online version contains supplementary material available at 10.1186/s12934-023-02121-2.

## Background

Steviol glycosides are high-sweet (300 times sweeter than sucrose), low-calorie natural sweetener extracted from the leaves of *Stevia rebaudiana* [1, 2]. Steviol glycosides also has good stability, has no effect on blood glucose, is non-fermentable, prevents caries, has no Browning reaction, and has therapeutic benefits [3–8]. It has recently gained popularity among consumers and developers worldwide. Since 2008, steviol glycosides has been approved by the World Health Organization Joint Expert Committee on Food Additives and other official organizations as a food and drug additive [9]. The main components of steviol glycosides are stevioside (ST) and rebaudioside A (RA) [10, 11]. Unfortunately, the bitterness of RA and ST is detected by half of the human population [12]. Enzyme-modified stevia (glucosylated steviol glycosides) emerged as a result of advancements in gene and protein engineering technology. Compared with steviol glycosides, its taste is closer to sucrose. Enzymatic modification of steviol glycosides refers to the use of enzymes to glycosylate steviol glycosides, that is, to attach the glucosyl groups to the C19 and/or C13 sites of steviol glycosides, which are crucial for taste. Transglycosylating enzymes, such as UDP-glucosyltransferase [13, 14] and cyclodextrin glucanotransferase (CGTase) [15–22], can perform this function. However, UDP-glucosyltransferase is rarely used in industry because it requires expensive nucleotide-activated sugars as glycosyl donors [23]. As a result, CGTase is commonly used in steviol glycoside modification (EC 2.4.1.19). CGTase is an enzyme of the glycoside hydrolase (GH) 13 family, that catalyzes the formation of starch to cyclodextrin through intramolecular transglycosylation [24]. In addition, CGTase catalyzes cyclization, coupling, disproportionation, and hydrolysis reactions [25], in which coupling and disproportionation play key roles in intermolecular transglycosylation. CGTase also has weak starch hydrolysis activity, it can be used to transglycosylate from donor substrates to various acceptor compounds, such as sugars, alcohols, vitamins, glycosides (such as steviol glycosides), polyols, and flavonoids, to improve their properties [25].

However, CGTase has poor specificity. For example, CGTase catalyzes the production of glucosylated steviol glycosides from steviol glycosides. CGTase can catalyze the production of mixtures of α glucosylated steviol glycosides (mono- to multiple-(α1–4)-glucosylated products) [15]. Some α glucosylated steviol glycosides have good taste and some have poor taste.

Notably, the efficiency of enzymes used in modification and the specificity of products greatly affect the quality of glucosylated steviol glycosides. In the existing technology, glucosylated steviol glycoside manufacturers mostly use CGTase-Toruzyme® 3.0 L produced by Novozymes, which makes the product category fixed and has a single taste. Therefore, mining a new efficient CGTase for enzyme modification of steviol glycosides is urgently needed.

From the product viewpoint, glucosylated steviol glycosides transformed with RA as the substrate have a better taste. In addition, currently known compounds with good taste are glucosylated on the basis of RA, and the “degree of glucosylation” is low [26–28], for instance, rebaudioside D (RD, add one glucosyl group on C19 of RA) and rebaudioside M (RM, add one glucosyl group on C19 of RD). Therefore, for the enzymatic modification of steviol glycosides, improving the conversion rate of RA and increasing the content of short-chain glycosylated products is of great importance for taste improvement.

In previous studies, CGTase was mainly engineered for the following purposes: (i) enhancing its heterologous or soluble expression [29]; (ii) increasing the specificity of the product, mostly commonly the specificity of α-, β-, or γ-cyclodextrin products [24, 30–32]; (iii) adjusting the levels of hydrolysis, cyclization, disproportionation, and coupling activities [33–36]; (iv) increasing the stability of CGTase [37–39]; and (v) relieving product inhibition [40, 41].

The objective of this work was to mine new CGTase, and improve the sensory profiles of its glycosylated steviol glycosides products through protein engineering. Using mass spectrometry-based secretome profiling, a novel CGTase for steviol glycoside modification was identified, and two sites with important roles in the conversion of steviol glycosides to glucosylated steviol glycosides were identified using site-directed mutagenesis. These two sites play crucial roles in increasing the conversion rate of RA and increasing the short-chain glycosylated product content, respectively, and they are also universal in other CGTases.

## Results and discussion

### Identification, heterologous expression, and characterization of a novel cyclodextrin glucosyltransferase

A CGTase-producing strain, *Alkalihalobacillus oshimensis* (also be named as *Bacillus oshimensis*), was isolated from *Stevia* planting soil in Shandong Province, China. Based on 16 S rRNA sequences, a phylogenetic tree was constructed (Additional file: Fig. S1a). Twelve possible CGTases were detected by LC-MS/MS, which were numbered 7–18. Based on amino acid sequences, a phylogenetic tree was constructed (Additional file: Fig. S1b) to verify the evolutionary relationship of the twelve possible CGTases (numbered 7–18) with six CGTases reported in the literature (numbered 1–6) [20–22, 26, 42, 43].

The 12 CGTases were expressed and purified in *E. coli*. Transglycosylation activity was analyzed in vitro. Among them, 5 CGTases exhibited relatively good transglycosylation activity. The best of the five was CGTase-13, followed by CGTase-8, CGTase-15, CGTase14, and CGTase-7 (data can be found in [44]).

**Fig. 1:**
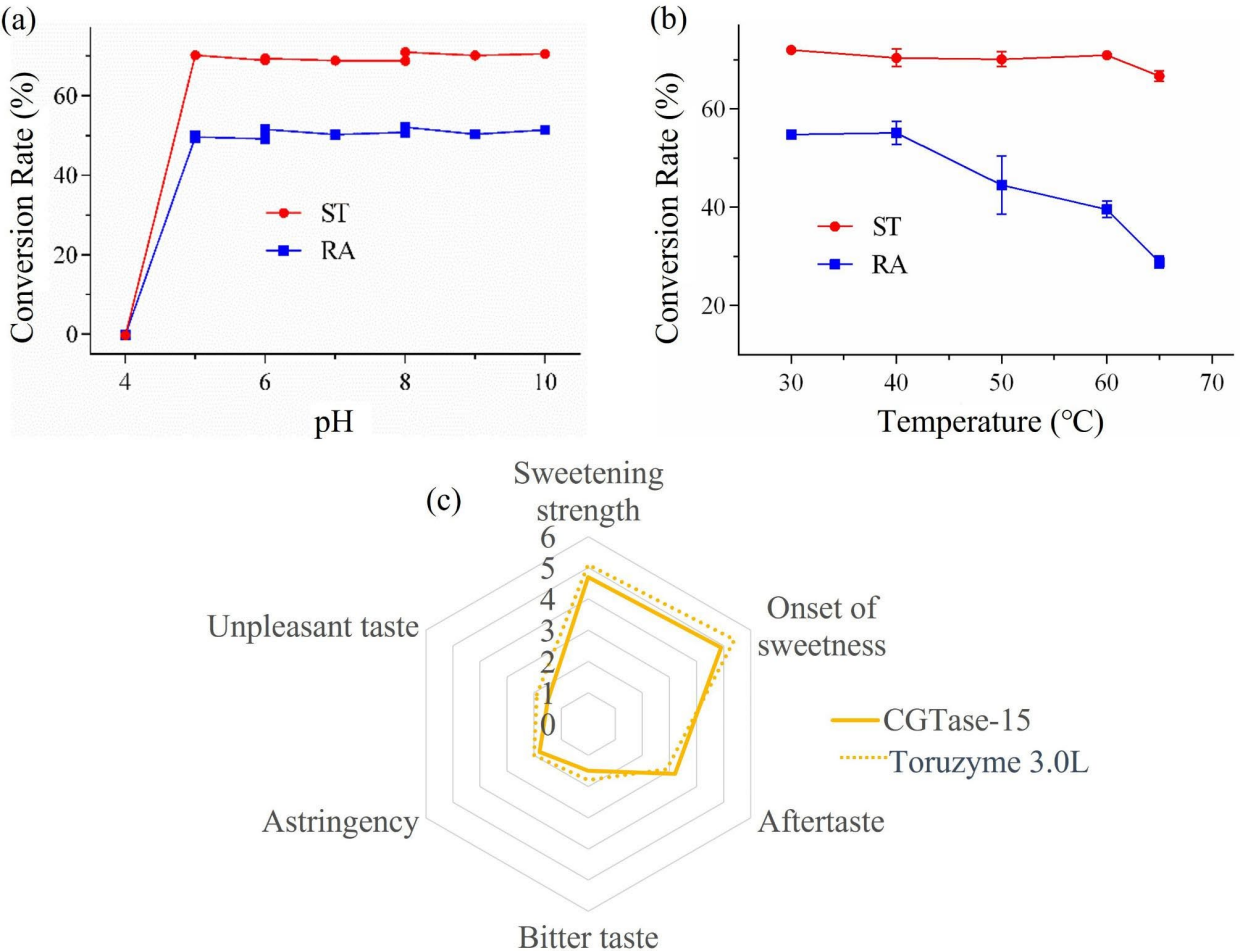
Effect of pH **(a)** and temperature **(b)** on the conversion rate of ST and RA to glucosylated steviol glycosides catalyzed by CGTase-15; **(c)** Radar-chart of sensory testing results (CGTase-15 and Toruzyme 3.0 L)

Through pH and temperature optimization, the optimum temperature of CGTase-15 was 30℃–40℃ and the optimum pH was 5–10 (Fig. 1a and b). The pH adaptation range of CGTase-15 was widest than other CGTase, which has certain significance for industrial application. So, CGTase-15 is taken as the research object.

To determine the type of CGTase-15, the α-, β-, and γ-cyclodextrins concentrations were detected. Results showed that the α-, β-, and γ-cyclodextrins concentrations were 0.203 ± 0.003, 0.300 ± 0.003 and 0.055 ± 0.000 mg/mL, respectively, which indicated that CGTase-15 belongs to β-CGTase, the most common type of CGTase used to modify of steviol glycosides.

The results of the sensory analysis are shown in Fig. 1c. Compared to Toruzyme® 3.0 L, the glucosylated steviol glycosides product produced by CGTase-15 had weaker bitter taste, astringency and unpleasant taste (Fig. 1c) and higher overall preference (data not shown). However, the steviol glycoside conversion rate and catalytic product taste of CGTase-15 are still need to be further improved, therefore, mutants of CGTase-15 were constructed.

### Construction of CGTase-15 mutants

To further improve the conversion rate of CGTase-15 to steviol glycosides and enhance the taste and quality of the product, site-directed mutagenesis of CGTase-15 was performed. Compared with steviol glycosides, an improvement in the quality was observed for the C-13 mono- and di-glucosylated products, while sweetness decreased for the C-13 tri-glucosylated product [20, 26]. Therefore, a high degree of glucosylation will also affect the taste and quality of the catalytic products (glucosylated steviol glycosides). As the main components of steviol glycosides are stevioside (ST) and rebaudioside A (RA) [10, 11], the detection indexes to evaluate the advantages and disadvantages of mutants were determined as the conversion rate of ST and RA to glucosylated steviol glycosides and the content of short-chain glycosylated products [20, 21, 26]. Amino acid residues near the substrate binding sites were preliminarily selected for mutation. Seven mutants that may affect transglycosylation activity, substrate selectivity, and product specificity were constructed as previously described: E150A [24], T151P [24], Y199F [45–47], E261G [48], G265A [49], L285M [50], and L285F [50] (Additional file: Fig. S2).

**Fig. 2:**
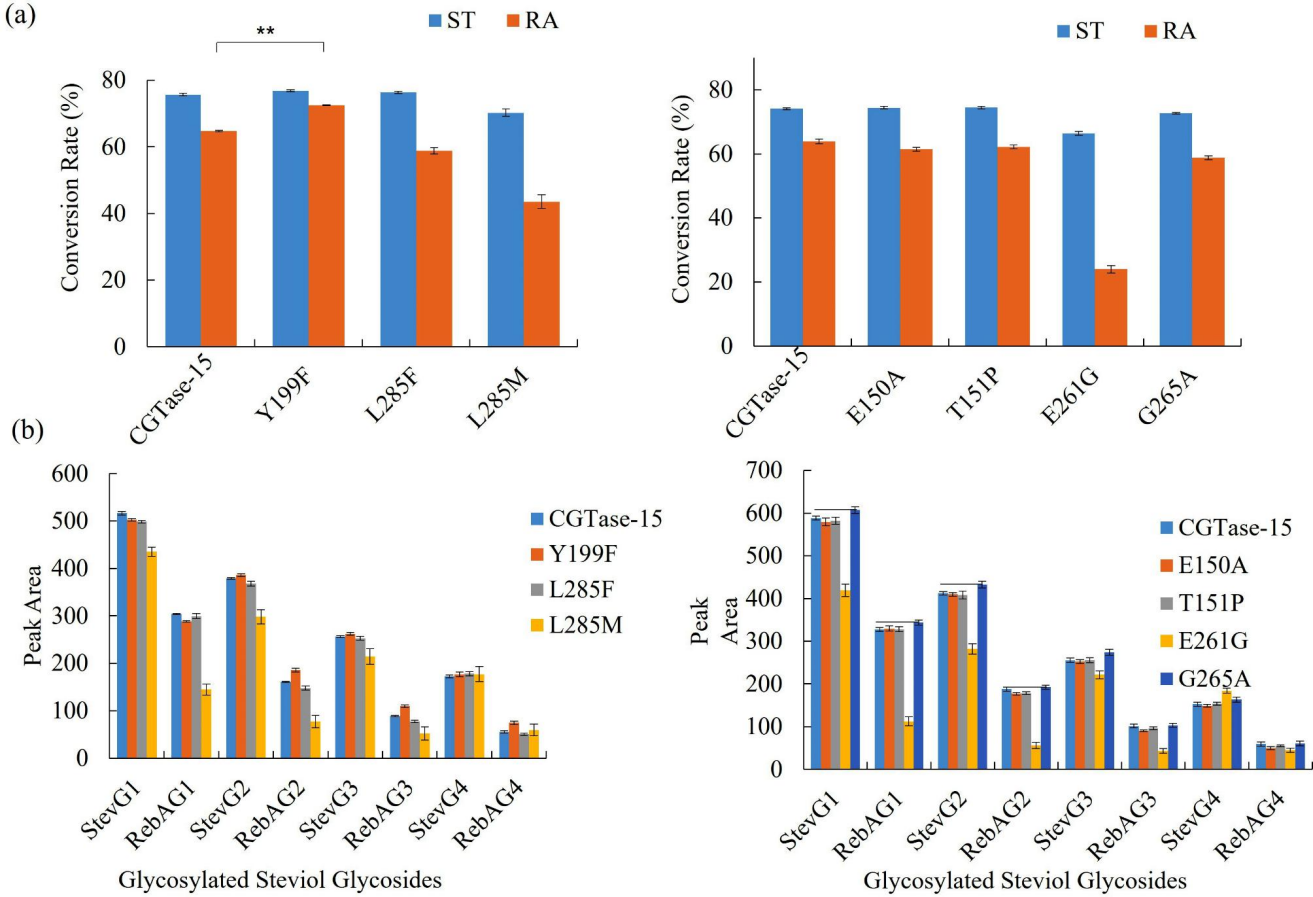
**(a)** Conversion rate of ST and RA to glucosylated steviol glycosides catalyzed by CGTase-15 and mutants; **(b)** HPLC analysis of modified steviol glycosides catalyzed by CGTase-15 and mutants

The conversion rates of ST and RA of these 7 mutants were tested. Notably, CGTase-15-Y199F mutant significantly increased RA conversion rates but did not change those of ST, whereas other mutants showed no significant change or decreased the conversion rates of ST and RA compared with that of the wild type (Fig. 2a). By analyzing the products, the content of the short-chain glycosylated product of the CGTase-15-G265A mutant increased although the conversion rate decreased slightly, whereas no obvious change was found for other mutants (Fig. 2b). Therefore, CGTase-15-Y199F and CGTase-15-G265A mutants were selected for further study.

### Effects of CGTase mutation on conversion rates of ST and RA and the content of short-chain glycosylated products

Firstly, CGTase-15-Y199F mutant was studied. To explore the probable binding modes of RA, structures of CGTase-15 and CGTase-15-Y199F mutant were constructed via homology modeling and, RA was used for docking. Molecular docking of RA was performed on the active site of CGTase-15/CGTase-15-Y199F mutant via the MOE docking program. To create the ligand–protein interaction plots for RA-CGTase-15-Y199F mutant/RA-CGTase-15, the MOE Ligand Interactions module was used, which provided a clearer arrangement of putative key intermolecular interactions.

**Fig. 3:**
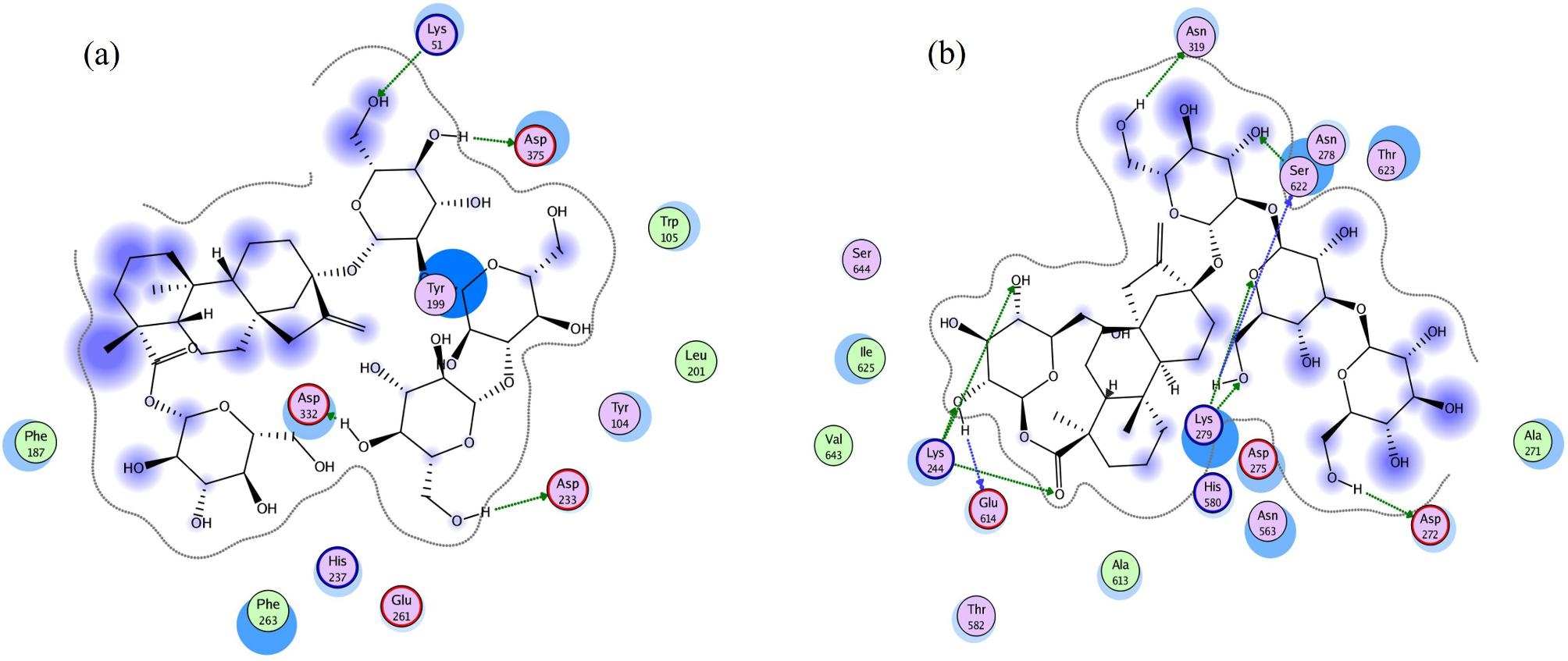
The two-dimensional interaction images of RA with CGTase-15 **(a)** and CGTase-15-Y199F mutant **(b)**

As shown in Fig. 3, CGTase-15-Y199F mutant exhibited better interaction with RA than CGTase-15. In CGTase-15-Y199F mutant, RA had nine interactions with the residues Lys244, Asp272, Lys279, Asn319, Glu614, and Ser622, whereas it had four interactions with the residues Lys51, Asp233, Asp332, and Asp375 in CGTase-15. The predominant bond was a hydrogen bond, implying a strong interaction of RA with CGTase-15 and CGTase-15-Y199F mutant.

**Fig. 4:**
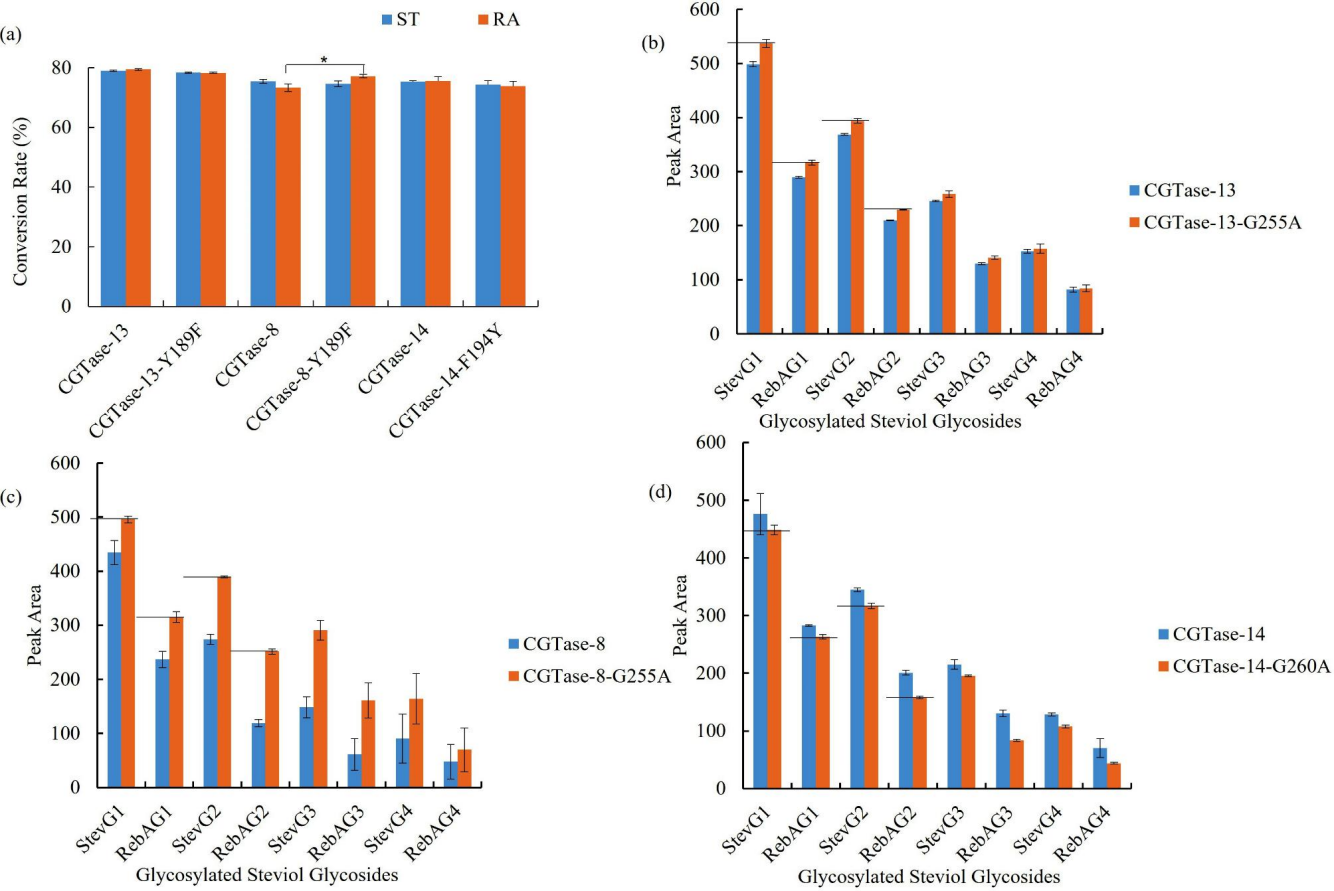
**(a)** Conversion rate of ST and RA to glucosylated steviol glycosides catalyzed by CGTases and their mutants (The control and test groups were compared using t-test. A p-value of < 0.05 was considered statistically significant: *p < 0.05, **p < 0.01, ***p < 0.001); HPLC analysis of modified steviol glycosides catalyzed by CGTases and their mutants (**(b)** CGTase-13 and CGTase-13-G255A mutant, **(c)** CGTase-8 and CGTase-8-G255A mutant, **(d)** CGTase-14-G260A mutant)

To test whether the mutation would show the same function in other CGTases, the same mutants of CGTase-8 and CGTase-13 were constructed, namely, CGTase-8-Y189F mutant and CGTase-13-Y189F mutant. Coincidentally, the corresponding residue in CGTase-14 was phenylalanine (F), therefore, CGTase-14-F194Y mutant was constructed for reverse verification. As shown in Fig. 4a, compared with CGTase-8, CGTase-8-Y189F mutant significantly increased the RA conversion rate. Compared with CGTase-13, CGTase-13-Y189F showed no significant change in the conversion rates of ST and RA. This may be because the RA conversion rate of CGTase-13 is inherently high, which is difficult to further improve. Compared with CGTase-14, CGTase-14-F194Y decreased the RA conversion rate but did not change that of ST (Fig. 4a). These results demonstrated the importance of this site in RA conversion.

Next, the CGTase-15-G265A mutant was studied. To test whether the mutation would show the same function in other CGTases, the corresponding mutants of CGTase-8, CGTase-13 and CGTase-14 were constructed, resulting in CGTase-8-G255A mutant, CGTase-13-G255A mutant, and CGTase-14-G260A mutant. As shown in Fig. 4b and c, compared with CGTase-8 and CGTase-13, both CGTase-8-G255A mutant and CGTase-13-G255A mutant increased the short-chain glycosylated product content in glucosylated steviol glycoside products. Interestingly, the results for CGTase-14-G260A mutant were completely opposite to those above. The content of short-chain glycosylated products catalyzed by CGTase-14-G260A mutant was lower than that of CGTase-14 (Fig. 4d). The low sequence identity (the sequence identities of CGTase-14 to CGTase-8, CGTase-13, and CGTase-15 were 55%, 55%, and 54%, respectively) and the long phylogenetic distance between CGTase-14 and the other three CGTases (Additional file: Fig. S1b) may result in the inapplicability of this universality in CGTase-14. However, the above characteristics proved that this site had an important effect on the content of short-chain glycosylated products in glucosylated steviol glycoside products.

We also speculated and verified why the mutation of G265 to A could increase the content of short-chain glycosylated products in glucosylated steviol glycoside products. Theoretically, highly grafted products have priority of hydrolysis [20, 50]. So, high hydrolytic activity might reduce the yield of highly grafted glucosylated steviol glycosides [20, 50]. However, by comparing the hydrolytic activity of CGTase-13-G255A mutant with CGTase-13, and CGTase-15-G265A mutant with CGTase-15, the hydrolytic activity of the mutants was lower than that of the wild type (Table 1), which was inconsistent with the literature reports and expectations [20, 50]. This indicates that the synergetic effects of different activities of hydrolysis, cyclization, coupling, and disproportionation may be the fundamental reason for the specificity of glucosylated steviol glycoside products. Notably, glucosylated steviol glycoside products are synthesized through intermolecular transglycosylation reactions (such as coupling and disproportionation) of CGTases, whereas long-chain products may be hydrolyzed or disproportionated to form short-chain products [20, 50].

**Table 1:** Specific activity of CGTases and their mutants

CGTase	activity (units/mg)
	hydrolysis
CGTase-13	4.8 ± 0.4
CGTase-13-G255A	2.2 ± 0.0
CGTase-15	2.8 ± 0.1
CGTase-15-G265A	2.2 ± 0.2

### Effects of CGTase mutation on the edulcorant quality of enzymatically modified steviol glycosides

As mentioned above, improving the conversion rate of RA and increasing the content of short-chain glycosylated products is of great importance for taste improvement. Considering the advantages of CGTase-15-Y199F and CGTase-15-G265A mutants compared to CGTase-15, the products catalyzed by the double mutant CGTase-15 may have better taste, so CGTase-15-Y199F/G265A mutant was constructed. Sensory analysis was performed to compare the tastes of modified steviol glycoside products catalyzed by CGTase-15-Y199F/G265A mutant and CGTase-15, respectively.

**Fig. 5:**
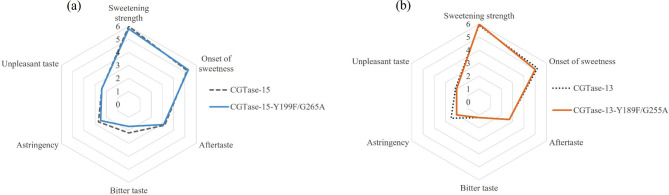
Radar-chart of sensory testing results (**(a)** CGTase-15 and CGTase-15-Y199F/G265A mutant, **(b)** CGTase-13 and CGTase-13-Y189F/G255A mutant)

**Fig. 6:**
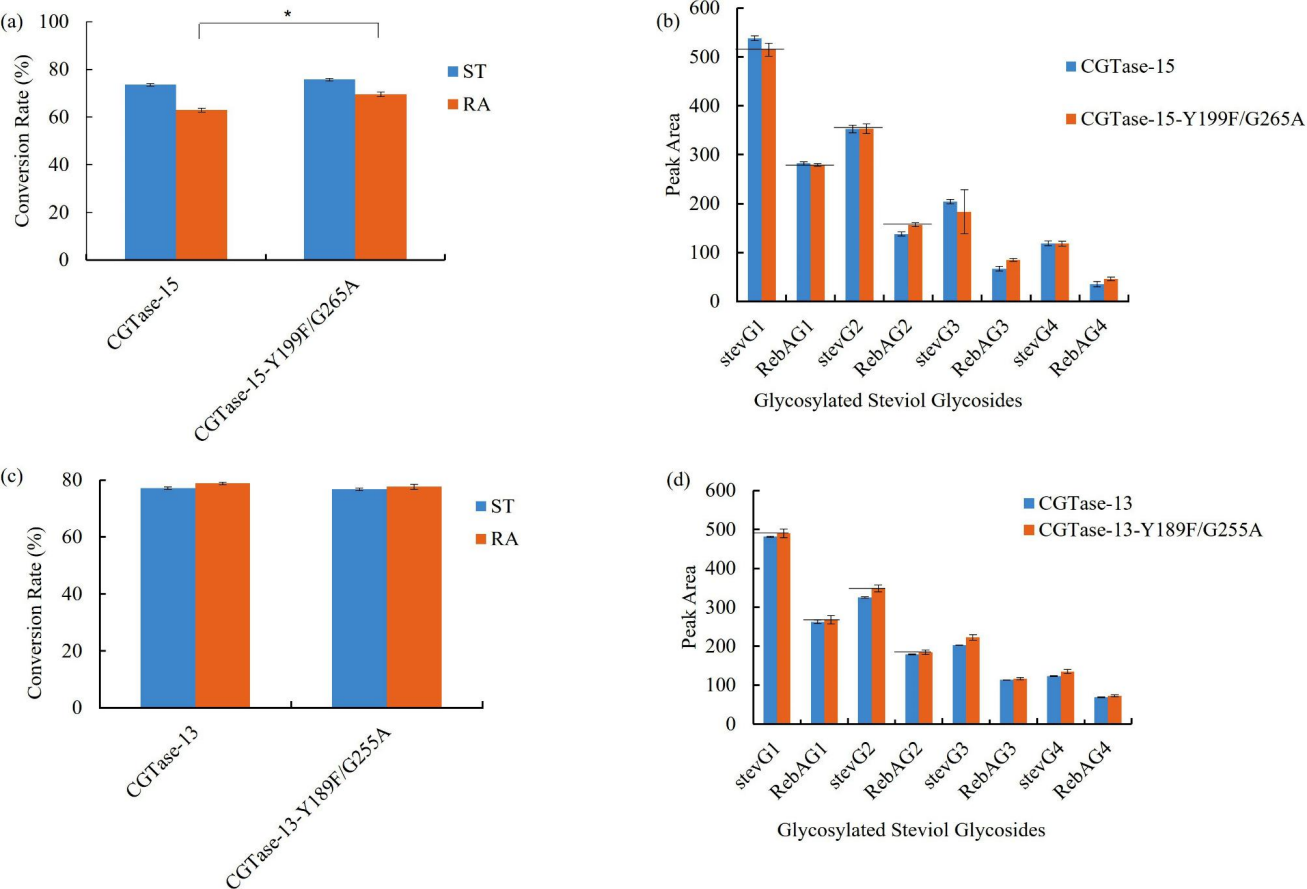
**(a)** Conversion rate of ST and RA to glucosylated steviol glycosides catalyzed by CGTase-15 and CGTase-15-Y199F/G265A mutant (The control and test groups were compared using t-test. A p-value of < 0.05 was considered statistically significant: *p < 0.05, **p < 0.01, ***p < 0.001); **(b)** HPLC analysis of modified steviol glycosides catalyzed by CGTase-15 and CGTase-15-Y199F/G265A mutant; **(c)** Conversion rate of ST and RA to glucosylated steviol glycosides catalyzed by CGTase-13 and CGTase-13-Y189F/G255A mutant; **(d)** HPLC analysis of modified steviol glycosides catalyzed by CGTase-13 and CGTase-13-Y189F/G255A mutant

The results showed that, compared to CGTase-15, the taste of the modified steviol glycoside products catalyzed by CGTase-15-Y199F/G265A slightly improved (Fig. 5a). The overall preference score of the catalytic products of CGTase-15-Y199F/G265A mutant and CGTase-15 were 70.8 and 70.3, respectively. We detect the conversion rates of ST and RA and the content of short-chain glycosylated products. The results showed that although the RA conversion rate was significantly increased after double mutation, the content of short-chain glycosylated products did not differ from that before mutation (Fig. 6a and b). So, the taste did not change much, indicating that improved RA conversion slightly affected taste, which was somewhat unexpected. To verify this conclusion, a comparison of the taste of modified steviol glycosides catalyzed by CGTase-15-Y199F mutant and CGTase-15, respectively, was conducted. The results showed that the taste of modified steviol glycosides catalyzed by CGTase-15-Y199F mutant was also not improved compared with CGTase-15 (data not shown). This result again show that the improvement of RA conversion rate has little effect on the taste.

And then, we studied the effect of the increase of short-chain glycosylated products on the taste. In this experiment, a double mutant, CGTase-13-Y189F/G255A mutant, was constructed. Note that CGTase-13-G255A mutant or CGTase-15-G265A mutant was not directly used because the conversion rate of steviol glycosides of CGTase-13-G255A mutant and CGTase-15-G265A mutant was slightly lower than that of CGTase-13 and CGTase-15. Fortunately, CGTase-13-Y189F/G255A mutant showed little change in the steviol glycoside conversion rate compared with CGTase-13 (Fig. 6c). This eliminates the effect of conversion rate. And compared with CGTase-13, the content of short-chain glycosylated products in the catalytic products of CGTase-13-Y189F/G255A mutant was increased (Fig. 6d), suggesting that the difference in taste in this experiment was primarily due to differences in the content of short-chain glycosylated products in the modified steviol glycosides.

The sensory analysis results revealed that CGTase-13-Y189F/G255A mutant improved the edulcorating quality of enzymatically modified steviol glycosides. Specifically, compared with CGTase-13, the glucosylated steviol glycoside product produced by CGTase-13-Y189F/G255A mutant had weaker astringency and unpleasant taste (Fig. 5b) and a higher overall preference. The overall preference score of the CGTase-13 catalytic product was 74.9, and that of CGTase-13-Y189F/G255A mutant was 77.8. This result corroborates previous reports that short-chain glycosylated products taste better than long-chain glycosylated products [20, 21, 26]. CGTase-13 is a CGTase discovered by our laboratory with great potential in the production of glycosylated steviol glycosides. The glucosylated steviol glycoside product produced by CGTase-13 exhibited excellent taste [44]. Through mutation, its potential in the production of glucosylated steviol glycoside was further improved.

Glucosylated steviol glycosides have great application potential, and controlling the degree of grafting to improve taste quality has also become a research hotspot. Previously, Li and colleagues controlled the degree of grafting in the glucosylated steviol glycoside product by controlling the reaction conditions, revealing that the content of mono- and di-glucosylated stevioside could be increased by slightly increasing the CGTase load and increasing the reaction temperature [20]. Similarly, Han et al. studied the relationship between long-chain glycosylated sophoricoside (LCGS) specificity and pH. The ratio of LCGS reached the highest level at pH 4, decreased to the lowest level at pH 5, and then gradually increased from pH 5 to 8 [50]. Son et al. synthesized stevioside having low degree polymerized glucosides using dextransucrase and dextranase [51]. In this study, we improve the taste quality of glucosylated steviol glycoside through the protein engineering of CGTase.

This study still has some shortcomings. First, short-chain glycosylated products are general term; Specifically to this experiment, they refer to mono-, di-glucosylated stevioside (StevG1 and StevG2), mono- and di-glucosylated rebaudioside A (RebAG1 and RebAG2). Second, compared with CGTase-15, CGTase-15-Y199F/G265A mutant increased the RA conversion rate from 62.9 to 69.6% with little change in taste, which could be due to insufficient conversion rate improvement. Finally, only hydrolytic activity was discussed in this paper, but the joint effects of cyclization, coupling, disproportionation, and hydrolysis were not extensively studied.

## Conclusion

In this study, a novel β-CGTase-CGTase-15, with a wide range of pH adaptation and catalytic product that tastes better than that of Toruzyme® 3.0 L, was identified from *A. oshimensis*. Two sites, Y199 and G265, which play important roles in the conversion of steviol glycosides to glucosylated steviol glycosides, were firstly identified by site-directed mutagenesis. By mutating Y199 to F, the RA conversion rate could be significantly increased, and by mutating G265 to A, the content of short-chain glycosylated products in glucosylated steviol glycoside products could be increased to improve the quality and taste of the product. The above findings have also been applied to CGTase-13, further increasing the potential of CGTase-13 in the production of glucosylated steviol glycoside. These findings are extremely important for the production of glucosylated steviol glycosides using CGTase and for the improvement of the sensory profiles of glucosylated steviol glycoside products.

### Experimental

#### Strain isolation and identification

The method of the strain isolation and identification was adapted from Yu et al. [22]. with small modifications. Briefly, on the basis of this method, we modified the dilution ratio and purified the strain several times. The 16 S rRNA gene sequence of the strain was registered in the GenBank nucleotide sequence databases with accession number OP060998. The isolated strain was then deposited at the China General Microbiological Culture Collection Center with strain number CGMCC 23,164.

#### Construction of phylogenetic trees

Phylogenetic trees were built with the neighbor-joining (NJ) algorithm within the Molecular Evolutionary Genetics Analysis program package (MEGA 7) [52]. The reliability of each branch was estimated with bootstrap replications (1000 replicates).

#### Protein identification using liquid chromatography-tandem MS (LC-MS/MS)

Protein identification was performed at the APTBIO. Protein bands (70–100 kDa) were cut from an SDS-PAGE gel. A gel trypsin digestion method was used before LC-MS/MS analysis were carried out. The peptides were separated using EASY nLC. Buffer solution comprised solution A (0.1% formic acid aqueous solution) and solution B (0.1% formic acid acetonitrile aqueous solution [84% acetonitrile]). The chromatographic column was equilibrated with 95% Solution A. Samples were loaded into the chromatographic column Thermo Scientific EASY column (2 cm × 100 μm; 5 μm-C18), followed by separation using the Thermo Scientific EASY column (75 μm × 100 mm; 3 μm-C18), with a flow rate of 300 nL/min. The liquid chromatography conditions were as follows: 1-h gradient; 0–50 min, linear gradient of solution B from 0 to 35%; 50–55 min, linear gradient of solution B from 35 to 100%; 55–60 min; solution B maintained at 100%.

Peptides analyzed by MS using Q-Exactive (Thermo Scientific). Full-scan MS spectra (*m/z* 300–1800) were obtained in positive ion mode. The mass spectra were searched against the Uniprot database (http://www.uniprot.org/) using MaxQuant.

#### Construction of mutants

The mutants were constructed using the Fast Mutagenesis System and Fast MultiSite Mutagenesis System of TransGen Biotech (Beijing, China), employing pET-28a (+)-CGTase/pPIC9K-CGTase as a template. Additional file: Table S1 in the supplementary information lists the sequences of primers. Clones were further confirmed by DNA sequencing, and the correct clones were transformed into *E. coli* BL21(DE3) or *Komagataella phaffii* for protein expression.

#### Heterologous expression and purification

The codon-optimized CGTase genes were synthesized and cloned in pET-28a (+) or pPIC9K via GENEWIZ (Suzhou, China). And then they were successfully expressed in *Escherichia coli* or *K. phaffii.* The nucleotide sequences of CGTases used in this study has been deposited in the GenBank database, the accession numbers are shown in Additional file: Table S2 in the supplementary information.

Heterologous expression and purification in *E. coli* refers to Zhang et al. [44].

Heterologous expression in *K. phaffii* refers to Zhang et al. [44].

The recombinant CGTases and their mutants were analyzed using SDS-PAGE. The results are shown in Additional file: Fig. S3.

#### Transformation of steviol glycosides into glucosylated steviol glycosides with CGTase

Steviol glycosides (2% (w/v)) and soluble starch (2% (w/v)) were dissolved in sterile water using as substrate. CGTase (20–21.2 mg/L) was added to the substrate solution, and the mixture was maintained at 28 to 65 °C for 24 h to conduct the reaction.

The reaction mixture was analyzed by HPLC (LC1200; Agilent Technologies, Inc., Santa Clara, CA, USA). The column used for screening strains is SB-C18 (4.6 × 250 mm; Agilent Technologies). The HPLC conditions are presented in Table 2.

**Table 2:** HPLC conditions

Time (min)	Acetonitrile (%)	Water (%)
0	25	75
5	25	75
30	50	50
34	50	50
35	25	75
40	25	75

Detection wavelength: 205 nm; sample injection volume: 40 µL.

Other transglycosylation reactions used Hypersil NH_2_ column (4.6 × 300 mm; Dalian Elite Analytical Instruments Co., Ltd., Dalian, China) for analysis. The HPLC conditions are presented in Table 3.

**Table 3:** HPLC conditions

Time (min)	Acetonitrile (%)	Water (%)
0	80	20
2	80	20
70	50	50
70.5	80	20
80	80	20

Detection wavelength: 210 nm; sample injection volume: 10 µL.

The conversion rates of ST and RA into glucosylated steviol glycosides were calculated as follows [20, 22]:

$$\text{S}\text{T} \text{c}\text{o}\text{n}\text{v}\text{e}\text{r}\text{s}\text{i}\text{o}\text{n} \text{r}\text{a}\text{t}\text{e} \left(\text{\%}\right)=({\text{C}}_{\text{o}}-{\text{C}}_{\text{t}})/{\text{C}}_{\text{o}}\ast 100\text{\%}$$

$$\text{R}\text{A} \text{c}\text{o}\text{n}\text{v}\text{e}\text{r}\text{s}\text{i}\text{o}\text{n} \text{r}\text{a}\text{t}\text{e} \left(\text{\%}\right)=({\text{C}{\prime }}_{\text{o}}-{\text{C}{\prime }}_{\text{t}})/{\text{C}{\prime }}_{\text{o}}\ast 100\text{\%}$$

where C_o_/C_o_′ is the initial ST/RA concentration, and C_t_/C_t_′ is the examinated ST/RA concentration after the reaction.

The content of each glucosylated steviol glycosides was determined according to its HPLC peak area. Example chromatograms before and after transglycosylation are shown in Additional file: Fig. S4.

#### Type determination

A soluble starch solution (2% (w/v)) prepared in 50 mM Na_2_HPO_4_/NaH_2_PO_4_ (pH 6.0) was incubated with the CGTase (20 mg/L) at 40 °C, 220 rpm for 18 h. Boiling water bath for 10 min to terminate the reaction. The reaction mixture was diluted 10 times, filtered with a 0.22 μm membrane, and detected with evaporative light-scattering detector Alltech 3300 (1,000,254,412; Waters Corporation).

The α-, β-, and γ-cyclodextrins concentrations in the final sample were analyzed by UPLC using an Acquity BEH phenyl column (2.1 × 100 mm, 1.7 μm; Waters) eluted with a methanol/water ratio of 1:99 at 0.3 mL/min.

#### Effects of temperature and pH on the conversion rate

Steviol glycosides (2% (w/v)) and soluble starch (2% (w/v)) dissolved in 50 mM Na_2_HPO_4_/NaH_2_PO_4_ (pH 7.0) were incubated with the CGTase (20 mg/L) at different temperatures (30 °C, 40 °C, 50 °C, 60 °C, and 65 °C), 220 rpm for 24 h. The conversion rate of ST and RA into glucosylated steviol glycosides was measured and calculated as described above.

The following buffers were used to estimate the effect of pH on the conversion rate of ST and RA into glucosylated steviol glycosides: 50 mM sodium acetate/acetic acid (pH 4–6), 50 mM Na_2_HPO_4_/NaH_2_PO_4_ (pH 6–8), and 50 mM Tris-HCl (pH 8–9.8). Steviol glycosides (2% (w/v)) and soluble starch (2% (w/v)) dissolved in a different pH buffer were incubated with the CGTase (20 mg/L) at 30 °C, 220 rpm for 24 h. The conversion rate of ST and RA into glucosylated steviol glycosides was measured and calculated as described above.

#### Sweetness and taste evaluation

Sensory analysis was conducted by Zhucheng Haotian Pharmaceutical Co., Ltd. For details of the method, please refer to the paper by Zhang et al. [44].

The samples were provided as solutions and included enzymatically modified steviol glycosides (product transformed by CGTase-15, CGTase-15-Y199F mutant, CGTase-15-Y199F/G265A mutant, CGTase-13, CGTase-13-Y189F/G255A mutant, and Toruzyme® 3.0 L, respectively). Concentration was 500 ppm. All samples for sensory analysis were blind samples for the panelists.

#### Molecular docking

Homology modeling of CGTase-15 and CGTase-15-Y199F mutant was performed using SWISS-MODEL. Molecular docking was performed in MOE. RA was docked into the target site of the predicted homology model of the CGTase-15/CGTase-15-Y199F mutant using an MOE-Dock module. The top-ranked conformation of RA was used for a detailed study of binding mode.

#### Hydrolytic activity analysis

The analysis of hydrolytic activity was adapted from Kong et al. [36]. with slight modifications. Soluble starch (1 g) was dissolved into 90 ml KH_2_PO_4_–Na_2_HPO_4_ buffer (pH 6.0) and divided into 2 mL centrifuge tubes, 450 µL /tube. Then, 50 µL, 200 µg/mL CGTase was added into the tube. The mixture was incubated at 50 °C for 0.5, 1, 2, 3, 4, and 5 h to carry out the reaction. The reaction was terminated by boiling water bath for 10 min. DNS reagent (1 mL) was added into the reaction solution, mixed, and centrifuged. Thirty microliters of supernatant was added to the microplate with 200 µL water in the well to determine OD_540_. One unit of hydrolysis activity was defined as the amount of CGTase required to produce 1 mmol maltose per minute. All the hydrolytic activity data presented represents the means of three independent detections.

## Electronic supplementary material

Below is the link to the electronic supplementary material.

**Additional file 1**. **Fig. S1**. (a) Dendrogram showing the relationship between the 16 S rRNA gene sequences of the strain studied in this study and some 16 S rRNA gene sequences of *Bacillus* sp. type strains obtained from the GenBank database; (b) Phylogenic analyses of CGTases from different sources. **Fig. S2**. Seven amino acid residues were selected for mutation. **Fig. S3** (a) SDS-PAGE of CGTase-15 and CGTase-15 mutants expressed in *Escherichia coli*. (b) SDS-PAGE of CGTase-13, CGTase-8, CGTade-14, and their mutants expressed in *E. coli*. (c) SDS-PAGE of CGTase-13, CGTase-13 mutants, CGTase-15, and CGTase-15 mutants expressed in *Komagataella phaffii.***Fig. S4** Example chromatograms before (a) and after transglycosylation (b). **Table S1**. Primers used in this study. **Table S2**. The nucleotide sequences used in this study.

## Data Availability

All data generated or analyzed during this study are included in this published article and its additional files.

## List of abbreviations

CGTase: Cyclodextrin glucanotransferase
ST: Stevioside
RA: Rebaudioside A
StevG1: Mono-glucosylated stevioside
StevG2: Di-glucosylated stevioside
RebAG1: Mono-glucosylated rebaudioside A
RebAG2: Di-glucosylated rebaudioside A.
