# Supplementary material for: Engineering of cyclodextrin glycosyltransferase improves the conversion efficiency of rebaudioside A to glucosylated steviol glycosides and increases the content of short-chain glycosylated steviol glycoside
Source: Microb Cell Fact. 2023 Jun 14;22:113. doi: 10.1186/s12934-023-02121-2 (PMC10265904; doi:10.1186/s12934-023-02121-2)
Supplement: Supplementary file 1 — Additional file 1. Fig. S1. (a) Dendrogram showing the relationship between the 16 S rRNA gene sequences of the strain studied in this study and some 16 S rRNA gene sequences of Bacillus sp. type strains obtained from the GenBank database; (b) Phylogenic analyses of CGTases from different sources. Fig. S2. Seven amino acid residues were selected for mutation. Fig. S3 (a) SDS-PAGE of CGTase-15 and CGTase-15 mutants expressed in Escherichia coli. (b) SDS-PAGE of CGTase-13, CGTase-8, CGTade-14, and their mutants expressed in E. coli. (c) SDS-PAGE of CGTase-13, CGTase-13 mutants, CGTase-15, and CGTase-15 mutants expressed in Komagataella phaffii.Fig. S4 Example chromatograms before (a) and after transglycosylation (b). Table S1. Primers used in this study. Table S2. The nucleotide sequences used in this study. [file 12934_2023_2121_MOESM1_ESM.docx]

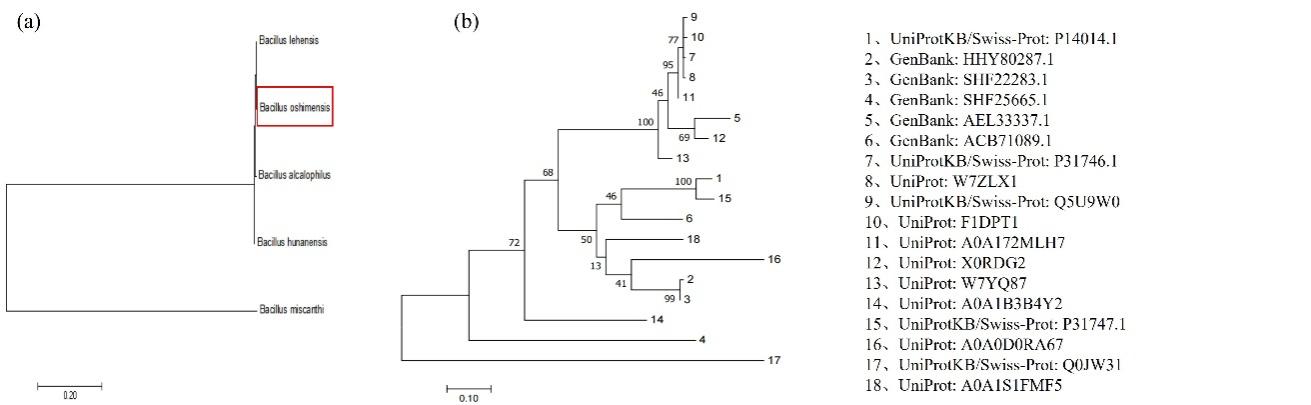


Fig. S1 (a) Dendrogram showing the relationship between the 16S rRNA gene sequences of the strain studied in this study and some 16S rRNA gene sequences of *Bacillus* sp. type strains obtained from the GenBank database; (b) Phylogenic analyses of CGTases from different sources


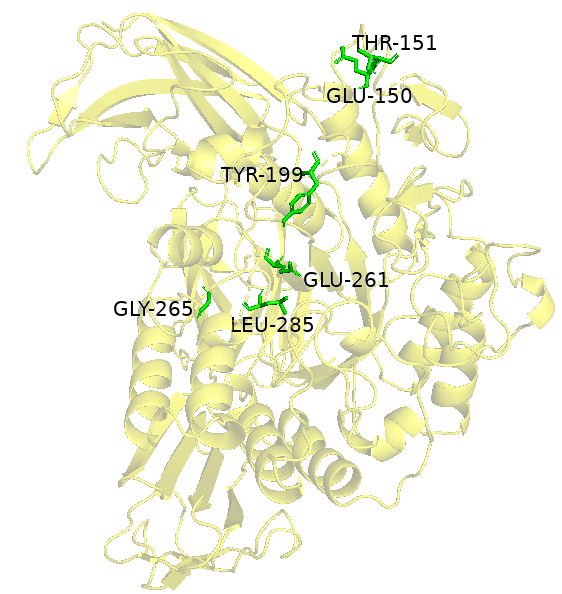


Fig. S2 Seven amino acid residues were selected for mutation


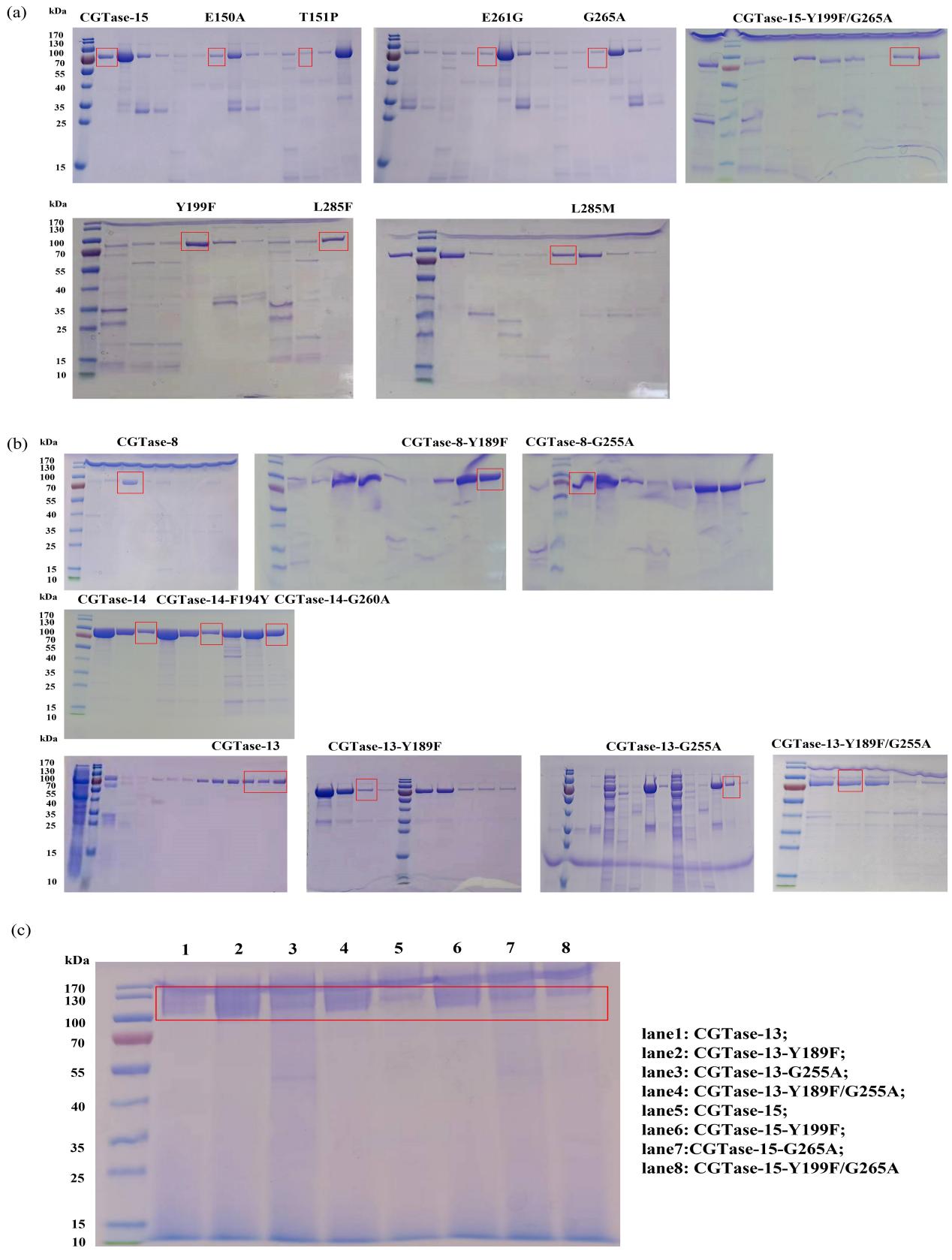


Fig. S3 (a) SDS-PAGE of CGTase-15 and CGTase-15 mutants expressed in *Escherichia coli*. (b) SDS-PAGE of CGTase-13, CGTase-8, CGTade-14, and their mutants expressed in *E. coli*. (c) SDS-PAGE of CGTase-13, CGTase-13 mutants, CGTase-15, and CGTase-15 mutants expressed in *Komagataella phaffii.*

*
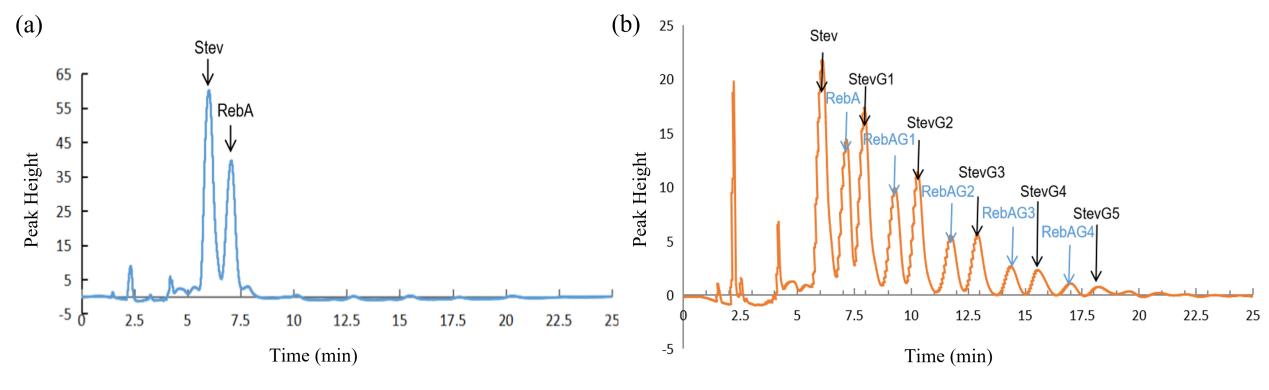
*

Fig. S4 Example chromatograms before (a) and after transglycosylation (b)

Table S1. Primers used in this study

| Primers | Sequences (5’-3’) |
| --- | --- |
| 15#-E-E150A-F: | TGGCGACCGATACGAGCTTTGCGGAAAACGGCAAAC |
| 15#-E-E150A-R: | TCCGCAAAGCTCGTATCGGTCGCCATCGCCGGGCTC |
| 15#-E-T151P-F: | AACCGGATACGAGCTTTGCGGAAAACGGCAAACTGT |
| 15#-E-T151P-R: | TTTTCCGCAAAGCTCGTATCCGGTTCCATCGCCGGG |
| 15#-E-Y199F-F: | CATTTATAAAAACCTGTTTGATCTGGCGG |
| 15#-E-Y199F-R: | AACAGGTTTTTATAAATGCCGTTTTCCAG |
| 15#-E-E261G-F: | GCGGCTGGTTTCTGGGCAGCGCGGCGAGCGATGCGG |
| 15#-E-E261G-R: | GCCGCGCTGCCCAGAAACCAGCCGCCAAAGGTAAAC |
| 15#-E-G265A-F: | TGGCGAGCGCGGCGAGCGATGCGGATAACACCGATT |
| 15#-E-G265A-R: | TCCGCATCGCTCGCCGCGCTCGCCAGAAACCATTCG |
| 15#-E-L285M-F: | AAAGCGGCATGAGCCTGATGGATTTTCGC |
| 15#-E-L285M-R: | TCAGGCTCATGCCGCTTTTGTTCGCAAAA |
| 15#-E-L285F-F: | TGTTTGATTTTCGCTTTAACAGCGCGGTGCGCAACG |
| 15#-E-L285F-R: | GCGCTGTTAAAGCGAAAATCAAACAGGCTCATGCCG |
| 15#-P-Y199F-F: | AATCTACAAGAACTTGTTTGATTTGGCTG |
| 15#-P-Y199F-R: | AACAAGTTCTTGTAGATTCCGTTCTCTAA |
| 15#-P-G265A-F: | TGGTGAATGGTTTTTGGCTTCTGCTGCTT |
| 15#-P-G265A-R: | GCCAAAAACCATTCACCAAAAGTAAAAAC |
| 13#-E-Y189F-F: | CATTTATCGCAACCTGTTTGATCTGGCGG |
| 13#-E-Y189F-R: | AACAGGTTGCGATAAATGCTATCTTCATA |
| 13#-E-G255A-F: | TGGCGAGCGGCGAAGTGGATCCGCAGAACCATCATT |
| 13#-E-G255A-R: | TGCGGATCCACTTCGCCGCTCGCCAGAAACCATTCG |
| 13#-P-Y189F-F: | TATTTGATTTGGCTGATTATGACTTGAACAATAAAG |
| 13#-P-Y189F-R: | AAGTCATAATCAGCCAAATCAAATAAGTTCCTATAA |
| 13#-P-G255A-F: | TGGTGAATGGTTTTTGGCTTCTGGTGAAG |
| 13#-P-G255A-R: | GCCAAAAACCATTCACCAAAAGTAAAAAC |
| 8#-E-Y189F-F: | TGTTTGATCTGGCGGACTATGATCTGAATAACACCG |
| 8#-E-Y189F-R: | AGATCATAGTCCGCCAGATCAAACAGGTTGCGATAA |
| 8#-E-G255A-F: | TGGCGAGCGGCGAAGTGGATCCGCAGAACCATCATT |
| 8#-E-G255A-R: | TGCGGATCCACTTCGCCGCTCGCCAGAAACCATTCG |
| 14#-E-F194Y-F: | TGTATGATCTGGCGAGCTTTAACCATATTAACCCGG |
| 14#-E-F194Y-R: | TGGTTAAAGCTCGCCAGATCATACAGGTTGCGATAA |
| 14#-E-G260A-F: | CCGCGCCGAACGGCAACGAAGATTATACCCGCTTTG |
| 14#-E-G260A-R: | TAATCTTCGTTGCCGTTCGGCGCGGTAAACCATTCG |

Table S2. The nucleotide sequences used in this study

| Names | Accession numbers |
| --- | --- |
| The Optimized (for *Escherichia coli*(*E.coli*)) sequence of 15-CGTase | OP095273 |
| The Optimized (for *Pichia pastoris*(*Yeast*)) sequence of 15-CGTase | OP095274 |
| The Optimized (for *Escherichia coli*(*E.coli*)) sequence of 13-CGTase | OP095271 |
| The Optimized (for *Pichia pastoris*(*Yeast*)) sequence of 13-CGTase | OP095272 |
| The Optimized (for *Escherichia coli*(*E.coli*)) sequence of 8-CGTase | OP095275 |
| The Optimized (for *Escherichia coli*(*E.coli*)) sequence of 14-CGTase | OP095277 |
